# How far will they go?: assessing the travel distance of current and former drug users to access harm reduction services

**DOI:** 10.1186/s12954-015-0043-4

**Published:** 2015-03-01

**Authors:** Sean Allen, Monica Ruiz, Allison O’Rourke

**Affiliations:** Department of Prevention & Community Health, Milken Institute School of Public Health at The George Washington University, 950 New Hampshire Ave, Suite 300, Washington, DC 20052 USA

**Keywords:** Syringe exchange programs, Access, People who inject drugs, Harm reduction

## Abstract

**Background:**

Prior research has explored spatial access to syringe exchange programs (SEPs) among people who inject drugs (PWID), but little is known about service utilization by former PWID who continue to access services (e.g., HIV screenings and referrals for social services) at harm reduction providers. The purpose of this research is to examine differences in access to SEPs between current and former PWID seeking services at a mobile SEP in Washington, DC.

**Findings:**

A geometric point distance estimation technique was applied to data collected as part of a PWID population estimation study that took place in Washington, DC, in March and April 2014. We calculated the walking distance from the centroid point of home residence zip code to the mobile exchange site where PWID presented for services. An independent samples *t*-test was used to examine differences in walking distance measures between current and former PWID. Differences in mean walking distance were statistically significant with current and former PWID having mean walking distances of 2.75 and 1.80 miles, respectively.

**Conclusions:**

The results of this study suggest that former PWID who are engaging with SEPs primarily for non-needle exchange services (e.g., medical or social services) may have decreased access to SEPs than their counterparts who are active injectors. This research provides support for expanding SEP operations such that both active and former PWID have increased access to harm reduction providers and associated health and social services. Increasing service accessibility may help resolve unmet needs among current and former PWID.

## Background

Existing research has empirically demonstrated the public health utility of syringe exchange programs (SEPs) for people who inject drugs (PWID). These programs are cost-effective, decrease the incidence of HIV among injectors, and have not been shown to increase drug use, crime, or presence of discarded syringes in neighborhoods [1-5]. While engaging with SEPs, PWID may experience benefits beyond provision of sterile injection equipment; for example, SEPs in the District of Columbia (DC) offer referrals to other services/programs (e.g., substance use treatment programs) and provide basic medical services (wound care, HIV/HCV screenings, etc.). For SEPs to be most efficacious, PWID must have easy access to their services. Research has shown that PWID who reside in close proximity to SEPs are more likely to consistently access services [6] and are less likely to share injection equipment [7].

Although research has quantified the distances between SEPs and areas of relevance to PWID (such as where substances are purchased/used and home residence) [8], these findings were based on data from 2002–2006 and may not be representative of current SEP access measures. It is plausible that SEP access changes overtime due to organizational closures, shifts in funding, or policy restrictions that limit SEP operations. SEP access may also vary by city.

For former PWID, it is important to maintain linkage with SEPs in order to access non-injection-related social and health services (e.g., referrals to housing or medical services) that may facilitate maintenance of sobriety and/or safer substance use practices if relapse occurs. No research has explored SEP access among PWID who are not actively injecting but who continue to utilize such services. This is an important gap in the literature given the likelihood of relapse among PWID and the need to maintain connections to health care resources [9]. The purpose of this research is to extend our knowledge of SEP access for PWID via two primary aims: (1) to provide updated estimates of distances that active injectors travel to access SEPs and (2) to quantify SEP access among former PWID who engage with SEPs for non-needle exchange services. We hypothesized that PWID in periods of non-active substance use would report shorter travel distances than their active injecting counterparts.

## Findings

### Methods

Data from a PWID population estimation study that used capture-recapture methodologies were used for this research. The population estimation study was divided into two 14-day periods of data collection with the first phase focused on collecting data among PWID who presented for services at SEP mobile distribution sites and the second phase focused on collecting data from PWID sampled from community venues (such as parks or other locations where PWID congregate). This study was conducted between March and April 2014 in partnership with two harm reduction service providers in DC. Only data from the largest mobile exchange program were used in this research as this site provides the bulk of syringes to DC PWID. Further, only data from the first phase of the population estimation study were used for this research, as this first phase of data collection sampled PWID who traveled to SEP sites to access services.

During the population estimation study, all individuals who presented for services received a verbal description of the study and were given the chance to ask questions about participation. If they verbally consented to participate, they were asked to complete an anonymous one-page survey that assessed individuals’ demographic characteristics, zip code of home residence, current frequency of substance use, methods of obtaining clean injection equipment, and drug use preferences. Small tokens of appreciation (such as toiletries kits or new socks) were given to each individual for their time in completing the survey. In efforts to maintain the anonymity of individuals presenting for services, the survey did not collect any personally identifying information.

Survey data were entered into an Access database by two members of the research team and a 10% random sample of surveys was checked for validation purposes. The locations of mobile exchange sites (public parks, intersections, shopping center parking lots, etc.) were geocoded using Google Maps [10]. Map data of zip codes in the United States were downloaded from the United States Census Bureau [11] and imported to ArcMap v10.2.1. ArcMap was used to calculate the latitude and longitude coordinates of the centroid point of each zip code. Centroid point data allowed for use of a geometric point distance estimation methodology to calculate walking distances between the centroid point of reported zip codes of home residence and exchange site. The geometric point distance estimation method is an analytic strategy in which one assumes that the centroid point of a given unit serves as the common origin for all data reporting that specific unit [12,13]. In this case, the unit refers to a zip code.

A SAS macro was created that quantified the walking distance between the two locations. Any instances where a person reported residing in a zip code outside of DC (thus indicating a non-DC resident) or at a post office box zip code (which may represent a location of convenience rather than a space near the participant’s home) were excluded from the analysis. Surveys with incomplete/missing information pertaining to substance use behaviors and zip code of home residence were also excluded from analysis. An independent samples *t*-test was used to determine if statistically significant differences existed in walking distance measures between persons who reported active substance use and those who reported no active substance use in the past 30 days at the time of engagement with the SEP.

This study was reviewed and approved by The George Washington University Institutional Review Board (IRB# 071315).

## Results

During the first phase of the population estimation study, 201 persons completed study forms. Among these, 152 reported residing in a valid geographic residential zip code in the District of Columbia. Fifteen forms were excluded due to missing information pertaining to injection drug use behaviors, resulting in a final sample of 137 PWID.

Overall, the majority (71%) of participants identified as male, African-American (78%), and with a mean age of 46.8 years. The independent samples *t*-test showed that the difference in mean walking distance between active and former PWID was statistically significant (*p* < .05), with active PWID having a mean walking distance of 2.75 miles and former PWID having a mean walking distance of 1.80 miles. These data are summarized in Table 1.

**Table 1:** **Data summary**

		**Overall (** ***n*** **= 137)**	**Active injectors (** ***n*** **= 97)**	**Non-active injectors (** ***n*** **= 40)**
Age (years)		46.8 (SD = 13.5)	47.4 (SD = 13.9)	45.6 (SD = 12.5)
Gender	Male	71% (*n* = 97)	68% (*n* = 66)	78% (*n* = 31)
	Female	25% (*n* = 34)	27% (*n* = 26)	20% (*n* = 8)
	Transgender	4% (*n* = 6)	5% (*n* = 5)	3% (*n* = 1)
	Missing	0% (*n* = 0)	0% (*n* = 0)	0% (*n* = 0)
Race	White/Caucasian	4% (*n* = 5)	5% (*n* = 5)	0% (*n* = 0)
	African-American/Black	78% (*n* = 107)	78% (*n* = 76)	78% (*n* = 31)
	Other	2% (*n* = 3)	0% (*n* = 0)	8% (*n* = 3)
	Missing	16% (*n* = 22)	16% (*n* = 16)	15% (*n* = 6)
Walking distance (miles)*		2.47 (SD = 2.10)	2.75 (SD = 2.23)	1.80 (SD = 1.59)

*p < .05.

## Conclusions

The results of this research show that PWID who are active and non-active injectors do not have equivalent access to SEP services. There are a few potential explanations for these findings. For example, PWID who are in periods of non-active substance use may have decreased motivation to travel to access non-injection-related services compared to when they were actively injecting. This explanation may be particularly valid if the main motivation for traveling to the SEP site was to obtain sterile injection equipment rather than other services. Another potential explanation is that non-active PWID may be seeking health care services elsewhere rather than going to the SEP site. We believe this explanation to be less valid given that, for many in this population, the mobile SEP unit is the only source of care that individuals have and/or trust. Harm reduction providers should consider these issues when selecting exchange sites.

Optimizing SEP access may increase retention and utilization of syringe exchange services among active PWID and increase access to social and health services that may help sustain substance use cessation, such as housing and other support services, for PWID who are not active injectors. Challenges in optimizing SEP service delivery are exacerbated by policies that restrict where SEP service provision may legally occur, such as those that prohibit SEP operations within certain proximities to schools [14]. Future work should explore how to enhance SEP service delivery in areas of greatest need while taking into account policy restrictions on service delivery and the non-equivalent access of current and former PWID to SEP services.

This study has a number of limitations that warrant discussion. The geometric point distance estimation method assumes that all persons who report a given unit of analysis—in this case, a zip code—reside at a geometric centroid point. This is an important limitation given that gentrification and changes in residential and commercial zoning may lead to uneven housing opportunities and population distributions within a given zip code. Further, we assumed persons are commuting from their home residence to the exchange site. It may be the case that PWID are coming to exchange sites from other points of origin, such as the places where they purchase or inject their drugs or work. Other research has explored the distance between these locations and SEPs [8], but none has explored this issue among PWID who are not in periods of active substance use.

Another limitation of this study is that we measured walking distance, which would provide an inaccurate estimate of travel distance if individuals were utilizing public or private means of transportation. While use of such modes of transport are a possibility, basing the travel estimate on walking distance was reasonable because our harm reduction partnering organizations in DC validated the fact that most of their PWID clients walk to access SEP services because they often cannot afford other means of transit. We cannot say with complete certainty that everyone in our sample walked to engage with the SEP. However, by limiting the analyses to persons who were recruited during the first phase of the study, we excluded persons who received services via delivery or secondary exchangers. Nonetheless, these walking distance estimates do not take into account other access barriers, such as time spent planning for and utilizing different modes of public transportation.

An additional limitation of this study is that it does not account for the range of factors that may influence SEP access, such as socioeconomic status or neighborhood characteristics. These data were not collected in the capture-recapture study because it was felt that the additional survey measures needed to obtain these data would pose undue burden on participants, thus making them reluctant to engage in our research. Future work should explore the range of factors that may influence PWID’s access to SEP and how these factors interact to affect consistent utilization of services.

Despite these limitations, these results highlight the important role of harm reduction organizations in meeting the needs of active and non-active PWID. Provision of comprehensive services for former substance users is important given this population’s vulnerability and their complex medical and social health care needs (such as housing and addiction treatment and support). Harm reduction providers are uniquely poised to address these needs, and can do so in a manner that will meet clients “where they are at” in their cycle of addiction and recovery.

## Abbreviations

PWID: Persons who inject drugs
SEPs: Syringe exchange programs
DC: Washington, DC
US: United States
